# Influence of Protonation and Iron(II) Complexation on Magnetic Interactions in Spin‐Labeled Mechanically Interlocked Molecules

**DOI:** 10.1002/chem.202500731

**Published:** 2025-04-25

**Authors:** Lorenzo Gualandi, Anna Turchetti, Paola Franchi, Lorenzo Sorace, Stephen M. Goldup, Fabio Canepa, Gianrico Lamura, Marco Lucarini

**Affiliations:** ^1^ Department of Chemistry “Giacomo Ciamician” University of Bologna Via P. Gobetti 85 Bologna 40129 Italy; ^2^ Department of Chemistry “Ugo Schiff” and UdR INSTM University of Florence Via della Lastruccia, 3 Sesto Fiorentino 50019 Italy; ^3^ School of Chemistry University of Birmingham Edgbaston Birmingham B15 2TT UK; ^4^ Department of Chemistry and Industrial Chemistry University of Genova via Dodecaneso 31 Genova 16146 Italy; ^5^ CNR‐SPIN Corso Perrone 24 Genova 16152 Italy

**Keywords:** ESR, molecular magnetism, rotaxane, supramolecular chemistry, spin labelling

## Abstract

This study reports the effects of protonation and Fe(II) complexation on the exchange coupling and magnetic properties of a [2]‐rotaxane and its corresponding free‐axle biradicals. The rotaxane features nitronyl‐nitroxide units as bulky stopper groups, a triazole‐pyridine‐triazole ligand as the axle component, and a bipyridine moiety within the macrocyclic ring. Our findings, obtained by Electron Paramagnetic Resonance spectroscopy in solution and magnetometry in solid state, demonstrate that rotaxanation and protonation induce profound changes in the magnetic interactions of the biradical both in its free form and as complexed with Fe(II). These results highlight the transformative impact of the mechanical bond on the electronic structure of interlocked radical systems.

## Graphical abstract

The study investigates the influence of protonation and Fe(II) complexation on exchange coupling and magnetic properties in a [2]‐rotaxane and its free‐axle biradicals. Mechanical bonding markedly modifies magnetic interactions, highlighting its potential for enabling the design of interlocked molecules with tunable spin states for advanced applications in molecular electronics and spintronics.

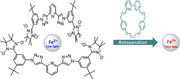

## 1 Introduction

Interlocked molecules,^[^ ^1^ ^]^ such as rotaxanes, exhibit distinctive physical and chemical properties that differ markedly from those of their individual covalent components. This unique behavior arises from the presence of the mechanical bond, a noncovalent interaction that imposes geometrical constraints on the system. The mechanical bond not only enables the creation of molecules with tailored reactivity and functional characteristics but also exerts a profound influence on the conformational dynamics of the covalent subcomponents. For instance, in the case of rotaxanes, the macrocyclic component is constrained from adopting conformations that would reduce its cavity to a size smaller than the axle it encircles. This conformational restriction can significantly affect the chemical and physical properties of the system.^[^ ^2^, ^3^ ^]^

In recent years, there has been a surge of interest in interlocked molecules containing persistent biradicals or polyradicals,^[^ ^4^ ^]^ driven by their potential applications in molecular machines.^[^ ^5^ ^]^ Numerous examples of such systems have been reported, including rotaxanes based on cyclophanes,^[^ ^6^ ^]^ cyclodextrins,^[^ ^7^ ^]^ cucurbiturils,^[^ ^8^ ^]^ octagonal metal rings,^[^ ^9^ ^]^ and crown ethers,^[^ ^10^ ^]^ where biradicals or polyradicals are incorporated as functional components. These radical centers serve a dual purpose: on one hand, they enable specific functionalities within molecular machines, ^[^ ^6a^ ^]^ and on the other, the mechanical bond itself can be leveraged to modify the intrinsic properties of the radical centers.

In 2021, some of us reported^[^ ^11^ ^]^ the synthesis and characterization of a novel family of rotaxane nitroxide^[^ ^12^ ^]^ biradicals featuring well‐defined conformational properties. These systems, prepared by using the active template approach,^[^ ^13^ ^]^ were based on a triazole‐pyridine‐triazole ligand^[^ ^14^ ^]^ as the axle component, functionalized with nitronyl nitroxide radicals at the bulky stopper units.^[^ ^15^ ^]^ Notably, rotaxanation has been shown to significantly alter spin‐spin exchange interactions (“*J*”) between radical centers compared to their unbound counterparts, offering a powerful tool for tuning the magnetic and electronic behavior of polyradicals.

The triazole‐pyridine‐triazole ligand is particularly notable for its ability to form metal complexes, while the pyridine nitrogen^[^ ^16^ ^]^ provides a convenient site for protonation under acidic conditions.^[14a,^ ^17^ ^]^ These features make the system highly dynamic and responsive to external stimuli, such as changes in pH or metal ion coordination.

**Scheme 1:**
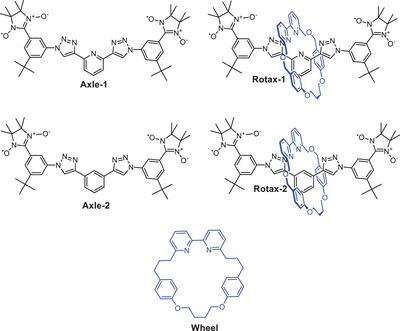
Structures of the *bis*(nitronyl‐nitroxide)s investigated.

In this study, we investigate the dynamic behavior of electron paramagnetic resonance (EPR)^[^ ^18^ ^]^ signals from both the free axle, **Axle‐1** (see Scheme 1), and the corresponding rotaxane, **Rotax‐1**, in response to variations in the local supramolecular environment.^[^ ^19^ ^]^ Specifically, we examine the effects of protonation and Fe(II) complexation on the electronic and magnetic properties of these systems. Our findings reveal that rotaxanation induces dramatic changes in the spin state of coordinated Fe(II) and leads to significant alteration in the conformational preference and consequently on the exchange coupling interaction in the biradicals. This observation underscores the transformative influence of the mechanical bond on the electronic structure of interlocked radical systems.

## 2 Results and Discussion

### 2.1 Effect of Protonation on Spin‐Exchange in **Axle‐1** and **Rotax‐1**

**Figure 1:**
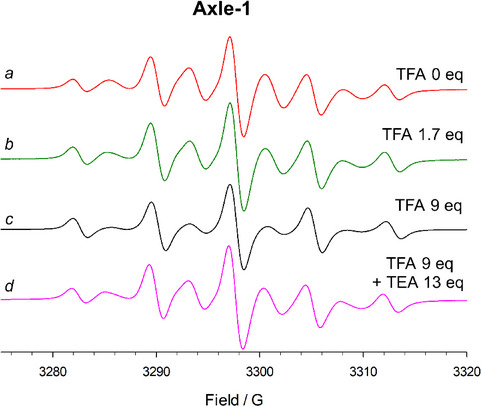
EPR titration of **Axle‐1** (0.3 mM) with TFA in MeCN at 298 K.

We initially investigated the EPR behavior of **Axle‐1** in the presence of trifluoroacetic acid (TFA), which is expected to protonate the central pyridine nitrogen atom. Figure 1 illustrates the EPR spectra of **Axle‐1** (0.3 mM), recorded at room temperature in MeCN, with progressively increasing concentrations of TFA.

To fully understand the EPR spectra, it is essential to remark that in the nitronyl‐nitroxide fragment, the coupling of the unpaired electron with two magnetically equivalent nitrogen atoms produces a characteristic five‐line signal with a relative intensity ratio of 1:2:3:2:1 with a line separation equal to hyperfine splitting constant *a*_N_. When the two chemically equivalent nitronyl‐nitroxide units within the biradical are strongly magnetically coupled through an exchange interaction ($\bar{J}$>>*a*_N_), the spectrum becomes more complex. This interaction introduces additional lines, resulting in a nine‐line pattern, where the separation between lines is determined by *a*_N_/2. Due to the weak delocalization of the unpaired spin onto the phenyl fragment, commonly observed in nitronyl‐nitroxides,^[^ ^20^ ^]^ and the 1,3‐substitution of the aromatic rings, which disrupts conjugation across the entire molecule, the through‐bond spin‐spin coupling can be considered negligible in **Axle‐1**. Consequently, the exchange interactions are primarily governed by through‐space effects, meaning they depend significantly on the specific conformation adopted by the biradical.

The EPR spectrum of **Axle‐1**, recorded at 298 K in MeCN (Figure  1a), reveals a pattern of nine lines with pronounced line width alternation. Based on the preceding discussion, this spectrum indicates a substantial average spin–spin interaction between the two radical units ($\bar{J}$>>*a*_N_).^[^ ^21^ ^]^ The observed line width alternation arises from the modulation of the exchange interaction by a slow interconversion between conformations with distinct radical‐radical distances.

When TFA is added to the solution containing **Axle‐1**, the spectra reveal a marked decrease in the intensities of the exchange lines, accompanied by a simultaneous increase in the intensity of the five‐line signal with rising TFA concentration (Figure 1b,c). The effect of pH on the EPR spectra was found to be fully reversible: the addition of an excess of triethylamine (TEA) restored the original spectral features, confirming the reversibility of the protonation process (Figure 1d).

As mentioned before, the observed EPR spectral behavior can be explained by the superposition of distinct conformational states, each characterized by differing spin exchange interactions. By analyzing the rotational flexibility of the bonds connecting the central aromatic ring to the two triazole units, three limiting conformations can be predicted for the 2,6‐*bis*(1,2,3‐triazol‐4‐yl)pyridine moiety.

**Scheme 2:**
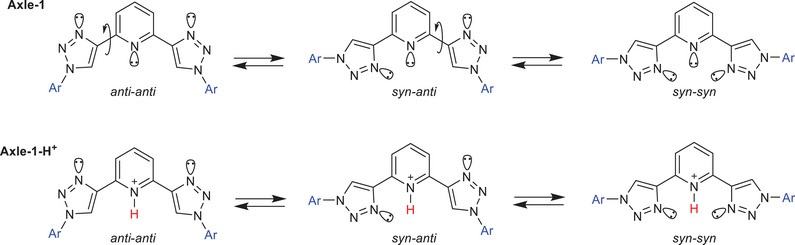
Conversion of the *anti‐anti* to the *syn‐syn* conformation in the free and protonoted 2,6‐*bis*(1,2,3‐triazol‐4‐yl)pyridine unit.

These conformations can be classified as either *syn* or *anti*, depending on the relative orientations of the triazole units (refer to Scheme 2). In the *anti‐anti* conformation, the two aryl fragments containing the radical centers are positioned relatively close to each other. In contrast, in the *syn‐anti* conformations, and even more so in the *syn*‐*syn* conformations, the two radical units are significantly farther apart. Actually, the average distances between the C2 carbons of nitronyl‐nitroxide units, estimated using Stochastic Dynamics (SD) simulations, were found to be greater than 13.7 Å in all *syn*–*syn* and *syn*–*anti* conformations, while in the *anti*–*anti* conformation, they can reach as short as 7.1 Å.^[^ ^11^ ^]^ As the value of *J* tends to be small when two N─O bonds interacting through space are separated by larger distances,^[^ ^4^, ^22^ ^]^ we can qualitatively predict that the exchange interaction between radical centers is negligible in all *syn* conformations, while it is appreciable/strong in the *anti‐anti* geometry.

The three conformations correspond to distinct energy levels.^[^ ^14^ ^]^ Notably, the 2,6‐*bis*(1,2,3‐triazol‐4‐yl)pyridine unit predominantly adopts an *anti*–*anti* conformation, which minimizes the repulsive interactions between the lone pairs of nitrogen atoms in the pyridine and triazole rings (Scheme 2). Computational studies indicate that the *anti* conformer is stabilized by 6.8 kcal/mol relative to the *syn* conformer.^[^ ^14^ ^]^

In conclusion, the EPR spectral behavior of **Axle‐1** observed in the presence of TFA can be explained by the superposition of two distinct spectral contributions: the first from *syn*–*syn* and *syn*–*anti* conformers (both with *J* = 0), and a second from *anti*–*anti* (with *J*≫a_N_). The relative contributions of these states are modulated by the protonation of the pyridine nitrogen, which mitigates the repulsive interactions between the lone pairs of adjacent nitrogen atoms (see Scheme 2) and produces favorable electrostatic attraction (hydrogen bond) between the triazole nitrogen lone pairs and the proton.^[^ ^17^ ^]^ Consequently, the addition of TFA to the solution is expected to drive a conformational switch in the triazole‐pyridine‐triazole ligand from the *anti*–*anti* state to the *syn*–*syn* state. This transition increases the distances between paramagnetic centers (see Scheme 2), leading to a decrease in the intensity of exchange lines in the EPR spectrum.

A final consideration pertains to the observation of two superimposed ESR spectra, which implies that the average lifetimes of the corresponding states are sufficiently long on the EPR timescale. Given that the rate of triazole‐phenyl‐triazole rotation is comparable to the EPR timescale, and assuming a similar behavior for the triazole‐pyridyl‐triazole ligand, the most plausible explanation is that proton transfer between the 2,6‐disubstituted pyridine and its conjugate acid is the rate‐limiting step (see Scheme 2). In the literature proton transfer from the 2,6‐disubstituted pyridinium ion to 2,6‐disubstitutedpyridine has been investigated by NMR line‐shape analysis both in a nonpolar (CD_2_Cl_2_) and polar (MeOH) medium.^[^ ^23^ ^]^ With 2,6‐dimethyl‐ and 2,6‐di‐*tert*‐butylpyridine at 298 K values of *k* = 1.1×10^3^ s^−1^ and *k* = 0.88×10^3^ s^−1^ have been reported, respectively. These values are significantly smaller than 10^5^–10^6^ s^−1^ representing the lower limit of rate exchange measurable by line width analysis in EPR.^[^ ^24^ ^]^ Thus, we can conclude that proton transfer between pyridyl and its conjugate acid is the rate limiting process.

For comparison, we also recorded the EPR spectrum of **Axle‐2** (see Scheme 1) in MeCN in the presence of TFA. In **Axle‐2**, the pyridine ring is replaced with a phenyl group. As expected, the absence of the basic pyridyl nitrogen prevents protonation, and the spectrum retained its original shape even after the addition of TFA (see Figure S1).

**Figure 2:**
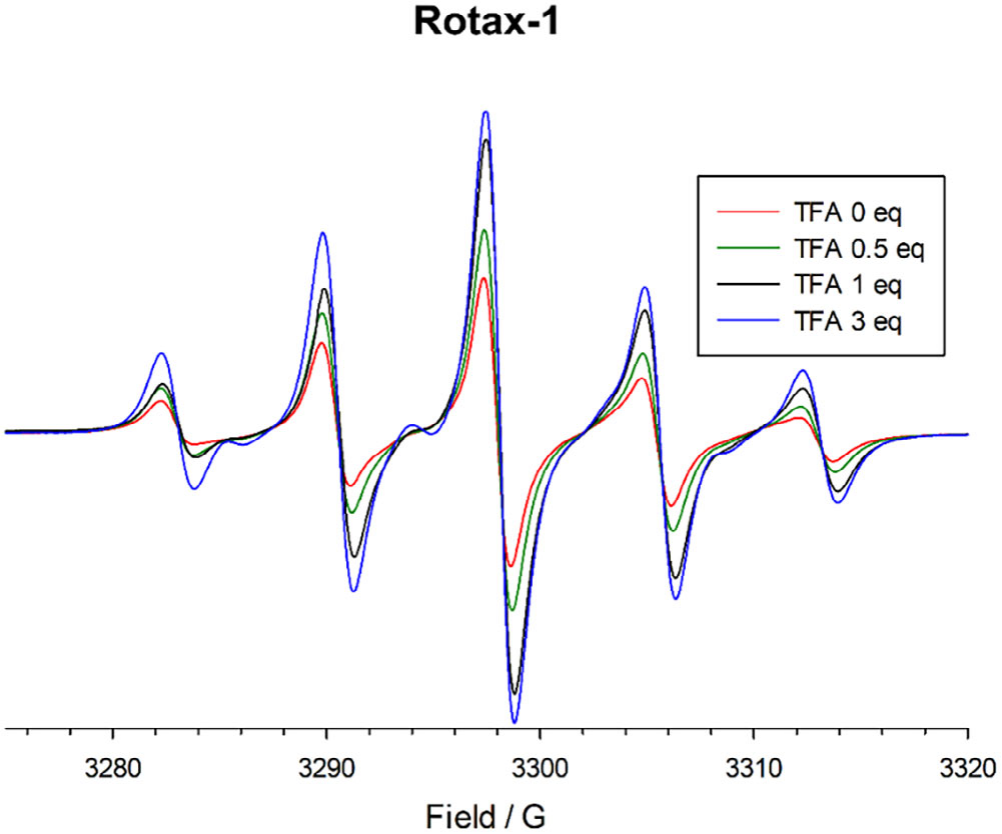
EPR titration of **Rotax‐1** (0.3 mM) with TFA in MeCN at 298 K.

We then investigated the combined effect of pH changes and rotaxanation on the EPR behavior of **Axle‐1** by measuring the EPR spectra of the corresponding rotaxane, **Rotax‐1**, in the presence of TFA (see Figure 2). The spectrum recorded in the absence of TFA at 298 K in MeCN displays only five lines. We already showed that the primary influence of the encircling macrocycle in the rotaxane is to slow the rate of conformational interconversion rather than to alter the strength of spin‐spin interactions.^[^ ^11^ ^]^ The mechanical bond imposed by the macrocycle reduces the interconversion rate between different conformations, leading to significant broadening of the lines due to exchange interaction, which consequently become undetectable.

As in **Axle‐1**, protonation of **Rotax‐1** is expected to increase the relative population of *syn‐syn* conformations, resulting in a more pronounced five‐line spectrum. Consistent with this expectation, increasing the concentration of TFA in MeCN enhanced the intensity of the EPR lines (Figure 2). Notably, the effect of pH on the EPR spectra was fully reversible: the addition of excess TEA restored the original spectral features, confirming the reversibility of the protonation process (Figure S2).

### 2.2 Metal Complexation of **Axle‐1** and **Rotax‐1**

As mentioned in the introduction, the triazole‐pyridine‐triazole motif is known to be a good ligand for metal cations^[^ ^14^ ^]^ as well as the bipyridine unit in the macrocyclic component of the [2]rotaxanes.^[^ ^13^ ^]^ Thus, we investigated the EPR properties of the *bis*(nitronyl‐nitroxide)s in the presence of Fe(II), a representative transition metal cation which can take two spin states [high spin (HS); S = 2 and low spin (LS); S = 0], depending on ligand field strength and external conditions.^[^ ^25^ ^]^

**Figure 3:**
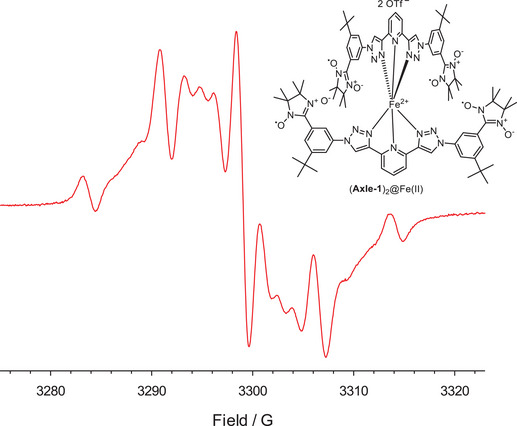
EPR spectrum of **Axle‐1** in the presence of Fe(OTf)_2_ in MeCN at 298 K.

Figure 3 presents the EPR spectrum of **Axle‐1** recorded in the presence of Fe(OTf)₂ in MeCN. The primary signal is characterized by a broad line centered at g = 2.0068, exhibiting a partially resolved hyperfine structure with separations of approximately one‐quarter of *a*_N_ (1.80 G). These findings strongly indicate the formation of a nitronyl‐nitroxide tetraradical complex (**Axle‐1**)_2_@Fe(II), where Fe(II) is in the low‐spin state and the four unpaired electrons of the radicals are hyperfine coupled to the eight nitrogen atoms of the nitronyl‐nitroxide groups. The proposed structure of this complex is analogous to that reported by Hecht and colleagues for a diamagnetic analogue (Figure 3).^[^ ^14^ ^]^

Reversible interconversion between the tetraradical and diradical forms was demonstrated by adding a large excess of 2,2`‐bipyridine (100:1) to a solution of the (**Axle‐1**)_2_@Fe(II) complex. Under these conditions, the EPR spectrum reverted to the original biradical signal (Figure S4).

**Figure 4:**
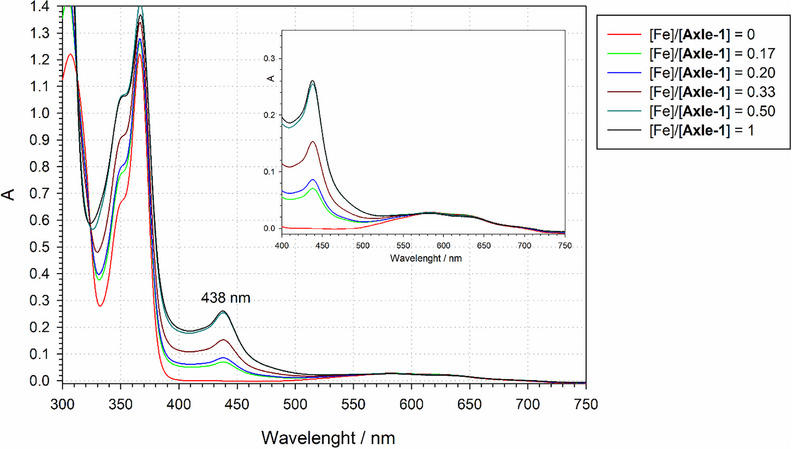
UV‐Vis titration of **Axle‐1** (0.058 mM) in MeCN at 298 K with Fe(OTf)_2_. Inset: expansion of the visible region.

The formation of the (**Axle 1**)₂@Fe(II) complex was further supported by UV‐Vis analysis in MeCN. Compared to the free axle, the absorption spectrum exhibits a distinct additional absorption band at 438 nm (see Figure 4) characterized by ε = 8849 l mol^−1^ cm^−1^ (Figure S8). Based on literature data on Fe(II) complexes of terpyridine, this band was attributed to metal‐to‐ligand charge transfer (MLCT) transitions in the complex.^[^ ^26^ ^]^

The 2:1 stoichiometry of the complex was confirmed through EPR and UV titration experiments by monitoring spectral variations upon incremental additions of Fe(II) to a solution of **Axle‐1**. Specifically, by EPR we tracked the intensity of the lines corresponding to the tetranitroxide complex (Figure S3), while by UV‐Vis spectroscopy we followed changes in the absorption band at 438 nm (Figure 4). Upon adding half an equivalent of Fe(II) relative to **Axle‐1**, no further spectral changes were observed in either experiment.

Definitive evidence of 2:1 stoichiometry was confirmed by recording the ESI‐MS spectrum of **Axle‐1** in the presence of a half‐equivalent of Fe(II) triflate (Figure S10). The main peak at *m/z* 815.5 exhibits signal separations of half *m/z* unit due to isotopomers, corresponding to a doubly charged species containing two **Axle‐1** units and one Fe(II) dication ([C_86_H_106_N_22_O_8_Fe]^2+^), as predicted from EPR and UV‐Vis analysis.

For comparison, was also recorded in the presence of Fe(OTf)₂ the EPR spectrum of **Axle‐2** in MeCN. As expected, the absence of the pyridyl‐coordinating nitrogen in **Axle‐2** prevented the formation of the tetraradical complex, and the spectrum retained its original shape (Figure S5).

**Figure 5:**
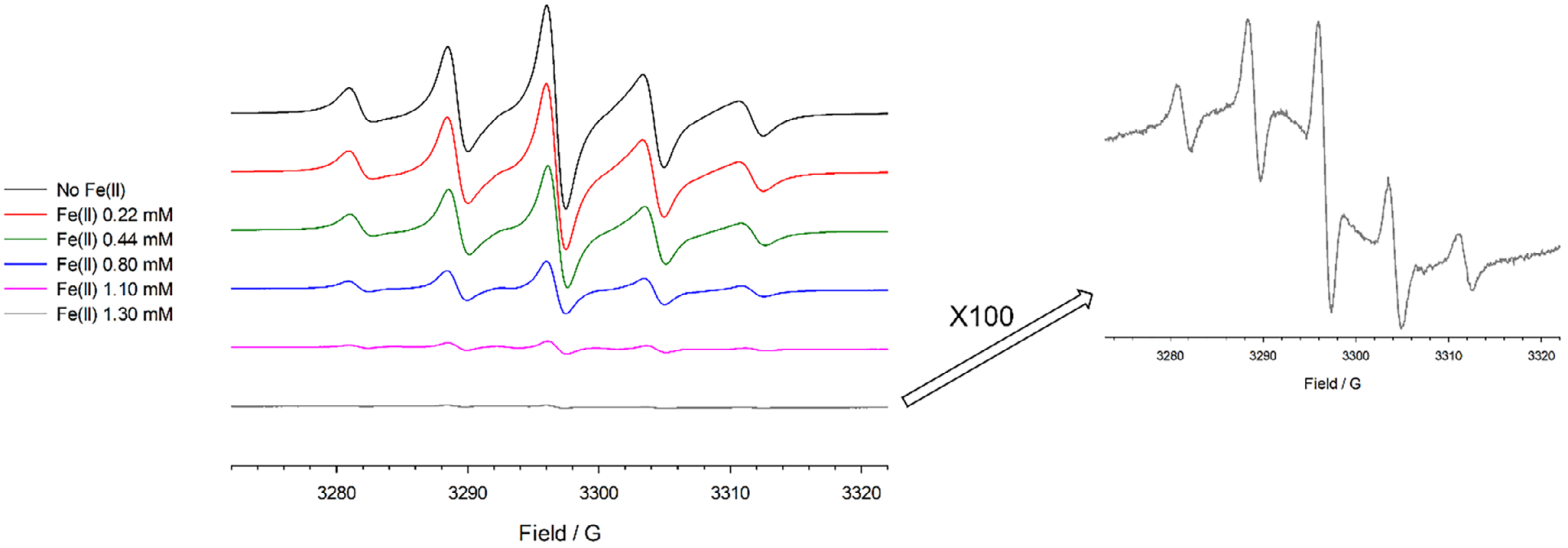
Left: EPR titration of **Rotax‐1** (1.1 mM) in MeCN at 298 K with Fe(OTf)_2_.On the right the spectrum in the presence of Fe(II) 1.5 mM is reported, recorded by increasing the gain 100 times.

A distinct EPR behavior was instead observed with **Rotax‐1** in the presence of Fe(OTf)₂. Incremental addition of Fe(II) to a solution of **Rotax‐1** resulted in a decrease in the intensity of the five‐line biradical signal and the emergence of a very broad signal (line width > 50 G) centered at g = 2.0068. This broad signal was detectable only by increasing the instrumental gain (see Figure 5). EPR titration, performed by adding increasing amounts of Fe(II), revealed that the five‐line signal was completely lost after the addition of 1 equivalent of Fe(II) (relative to the nitroxide). Notably, the double‐integrated EPR signal remained constant throughout the titration, indicating that the total number of radical species did not change upon Fe(II) addition. This is suggestive of Fe(II) forming high‐spin species in these conditions, which are well known to have large zero‐field splitting and thus being “EPR‐silent” at conventional frequencies.^[^ ^27^ ^]^ At the same time, these high‐spin species would be fast relaxing, thus explaining the broadening induced on the radical spectra.

Consistent with previous observations, the addition of 2,2`‐bipyridine restored the original spectrum of **Rotax‐1** (Figure S6). In contrast, no such broad signal was observed when Fe(II) was added to **Rotax‐2**, underscoring the critical role of the coordinating pyridyl nitrogen atom (Figure S7).

We attributed the observed EPR behavior to the formation of a new 1:1 complex (**Rotax‐1**@Fe(II)) in which rotaxanation changes Fe(II) from a diamagnetic low‐spin state (S = 0) to a paramagnetic high‐spin state (S = 2). Unfortunately, attempts to obtain suitable single crystals for X‐ray structure determination were unsuccessful. However, the HS configuration of the Fe(II), confirmed in the solid state (see below), points either to a weaker ligand field than in (**Axle‐1)_2_@Fe(II)** or to a different coordination geometry. This consideration together with the 1:1 stoichiometry determined for the complex and the comparison to similar metal‐rotaxane complexes^[^ ^28^ ^]^ suggests that the Fe(II) coordination might be trigonal bipyramidal rather than octahedral.

The UV‐Vis analysis of **Rotax‐1** (Figure S9) shows a significant change in the spectral shape of the absorption in the 300–450 nm region after the addition of Fe(II) triflate. However, the charge transfer band from the metal to the ligand overlaps with the absorption bands of the rotaxane's aromatic rings, preventing an accurate analysis.

In contrast, the ESI mass spectrum (Figure S11) displays a clear single intense peak at *m/z* 662.8, corresponding to the 1:1 dicationic complex between Fe and the rotaxane ([C_73_H_83_N_13_O_8_Fe]^2+^).

### 2.3 Magnetic Measurements

**Figure 6:**
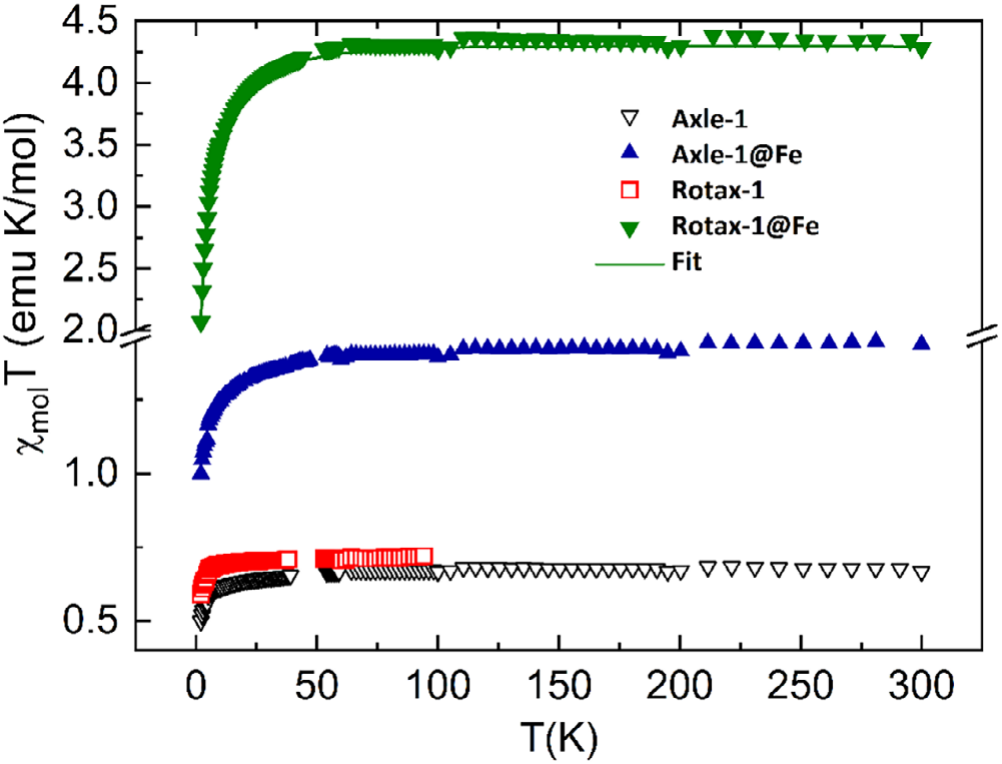
Temperature dependence of the χ_mol_T product for the four investigated derivatives. The continuous line represents the best fit to **Rotax‐1@Fe** data obtained with model and parameters described in the text.

In order to determine the spin state of Fe complexes in the solid state we performed magnetic measurements both on the free and Fe(II) complexed **Axle‐1** and **Rotax‐1**. Magnetic measurements of the four samples are reported as the product of the molar magnetic susceptibility (χ_mol_T) as a function of temperature (Figure 6), and their behavior provides some important clues about the solid‐state structure of the compounds and the interactions between the constituent paramagnetic moieties.

For **Rotax‐1** the measured χ_mol_T values up to 100 K are in good agreement with expectations for two independent S = 1/2 spins, based on Curie's law (χ_mol_T(exp) = 0.69 emu K mol^−1^, χ_mol_T(calc) = 0.75 emu K mol^−1^). The Curie‐Weiss plot (Figure S12) is linear in the whole temperature range with a negative Weiss temperature (C = 0.719 emu K mol^−1^, θ = ‐ 0.5 K), suggesting the presence of weak antiferromagnetic exchange interactions between radicals, either intermolecular or intramolecular. The small discrepancy between experimental and theoretical Curie constant values might be attributed to a partial degradation of the radical, in much the same way as observed for **Axle‐1** (Figure S12, C = ‐0.681 emu K mol^−1^, θ = ‐1.1 K). Even though in the solid state the role of intermolecular magnetic interactions cannot be neglected, for both derivatives the magnitude of the interactions as indicated by the Weiss temperatures is consistent with the results obtained by EPR investigation, being much larger than the hyperfine coupling constant.

At variance with the two organic derivatives, the measurements for the two Fe(II) complexes differ significantly from one another. The room temperature χ_mol_T value measured for **Rotax‐1@Fe** is as expected for a high‐spin Fe(II) center (g ≈ 2.1‐2.2, S = 2)^[^ ^29^ ^]^ and two radicals (χ_mol_T (exp) = 4.2 emu K mol^−1^ versus χ_mol_T(theo) = 4.05‐4.4 emu K mol^−1^), thus confirming the stoichiometry postulated on the basis of the EPR spectrum. The decrease observed in χ_mol_T on lowering temperature below 55 K can be in principle attributed either to the zero field splitting (ZFS) of the Fe(II) ion which is known to be relatively large^[^ ^27b^ ^]^ or to the presence of moderate exchange interaction between Fe(II) and the radicals. The latter hypothesis is however in contrast with the observed isothermal magnetization curves (Figure S13) measured at low temperature, which do not show evidence of this interaction.

The validity of this model was confirmed by simultaneously fitting^[^ ^30^ ^]^ the isothermal magnetization and the χ_mol_T versus T curves by assuming the following spin Hamiltonian and an intermolecular interaction correction term (*zJ*, see below):

$$\;\hat{H}=B\cdot \left(g_{\mathrm{Fe}}{\hat{S}}_{\mathrm{Fe}}+g_{\mathrm{rad}1}{\hat{S}}_{\mathrm{rad}1}+g_{\mathrm{rad}2}{\hat{S}}_{\mathrm{rad}2}\right)+D_{\mathrm{Fe}}{\hat{S}}_{z}^{2}+E_{\mathrm{Fe}}\left({\hat{S}}_{x}^{2}-{\hat{S}}_{y}^{2}\right)$$

where *D*_Fe_ and *E*_Fe_ are the axial and rhombic ZFS parameters, respectively.

Best fit results (*R* = $\left[\sum {\left(M_{\exp}-M_{\mathrm{calc}}\right)}^{2}\right]\left[\sum {\left(\chi_{\exp}-\chi_{\mathrm{calc}}\right)}^{2}\right]$ = 0.00396) were obtained by fixing *g*_Fe_ = 2.17, *g*_rad1_ = *g*_rad2_ = 2.00, and *D*_Fe_ = 16.2 ± 0.2 cm^−1^, *E*_Fe_ = 3.85 ± 0.05 cm^−1^. The calculated susceptibility was then corrected by an intermolecular correction term, *zJ* = 0.062 ± 0.003 cm^−1^ according to $\chi_{\mathrm{corr}}=\frac{\chi_{\mathrm{calc}}}{\left(1-\chi_{\mathrm{calc}}zJ/N_{A}\beta^{2}\right)}$. On the other hand, neglecting the intermolecular interaction term and including intramolecular exchange coupling interactions (either Fe(II)‐radical or radical‐radical ones) failed to provide an appropriate reproduction of the magnetic data. In the framework of the model used to reproduce the magnetic data it is also possible to rationalize the observed EPR spectrum for **Rotax‐1@Fe** (see Figure 5): intermolecular radical‐radical interactions and the proximity of the radicals to the largely anisotropic and fast‐relaxing Fe(II) centers broaden the radical spectrum to provide a featureless band centered at *g* = 2.00; the residual hyperfine structure observed is then to be attributed to a small amount of residual, uncomplexed radicals. The large ZFS and the integer spin state further makes Fe(II) silent at X‐band frequency.

Finally the χ_mol_T versus T observed for **Axle‐1@Fe(II)** can only be explained by assuming that in this complex Fe(II) is in its low‐spin configuration due to the strong ligand field of the coordinating molecules and is thus not contributing to the magnetic properties of the molecule. The room temperature value is then consistent with the presence of 4 radical spins S = 1/2 (χ_mol_T (exp) = 1.4 emu K mol^−1^ χ_mol_T(theo) = 1.5 emu K mol^−1^), again in agreement with the stoichiometry postulated by the analysis of the EPR and UV‐Vis spectra. The observed decrease at low T suggests further that weak antiferromagnetic interactions are present, a point which is confirmed by the fit of the Curie‐Weiss plot with best fit parameters C = 1.44 emu K mol^−1^, θ = ‐ 1 K (Figure S12)

## 3 Conclusions

We demonstrate that the mechanical bond allows access to complexes that are unavailable from the corresponding noninterlocked components. These results can have broader implications for understanding the behavior of biradical and polyradical systems under variable chemical conditions. By elucidating the interplay between the mechanical bond, radical centers, and external stimuli such as protonation and metal complexation, our work provides an insight into the design and functionality of interlocked molecules for advanced applications. The ability to fine‐tune spin states and exchange interactions of organic radicals through mechanical bonding opens exciting possibilities for the development of novel materials and devices in the fields of molecular electronics, spintronics, and beyond.^[^ ^31^ ^]^

## Conflict of Interests

The authors declare no Conflict of interest.

## Supporting information



Supporting Information

## Data Availability

The data that support the findings of this study are available in the supplementary material of this article.
