# Supplementary material for: Influence of Protonation and Iron(II) Complexation on Magnetic Interactions in Spin‐Labeled Mechanically Interlocked Molecules
Source: Chemistry. 2025 Apr 25;31(29):e202500731. doi: 10.1002/chem.202500731 (PMC12099185; doi:10.1002/chem.202500731)
Supplement: Supplementary file 1 — Supporting Information [file CHEM-31-e202500731-s001.pdf]

Supporting information for

Influence of Proton and Iron(II) Complexation on Magnetic Interactions in Spin-Labeled Mechanically Interlocked Molecules

Lorenzo Gualandi, Anna Turchetti, Paola Franchi, Lorenzo Sorace, Stephen M. Goldup, Fabio Canepa, Gianrico Lamura, Marco Lucarini

---

1. General Methods 2
2. EPR spectra and titrations 3
3. UV-vis spectra 6
4. ESI-MS spectra 7
5. Curie-Weiss plot 7
6. Isothermal magnetization curves 7

General Methods

Synthesis and characterization of nitroxides **Axle-1**, **Axle-2**, **Rotax-1** and **Rotax-2** have been previously reported [L. Gualandi, P. Franchi, E. Mezzina, S. M. Goldup, M. Lucarini, *Chem. Sci.*, 2021, 12, 8385].

Commercial reagents and solvents were used as received, without further purification. When necessary, solvents were degassed by bubbling nitrogen.

EPR spectra has been recorded on Bruker-ELEXYS spectrometer by using the following instrument settings: microwave power 0.79 mW, modulation amplitude 0.04 mT, modulation frequency 100 kHz, scan time 180 s, 2K data points.

ESI-MS spectra were recorded with Micromass ZMD spectrometer by using the following instrumental settings: positive ions; desolvation gas (N2) 230 L/h; cone gas (skimmer): 50 L/h; desolvation temp. 120° C; capillary voltage: 3.2 kV; cone volt-age: 40 and 100 V; hexapole extractor: 3 V.

UV-vis spectra were recorded in MeCN with Cary 3500 double beam spectrophotometer by using standard 1 cm path length quartz cell.

Magnetic measurements were performed using a MPMS SQUID magnetometer (Quantum Design, Inc.) on powder microcrystalline samples. Raw data were corrected for the diamagnetism of the sample holder and the intrinsic diamagnetic contribution of the sample, estimated by Pascal's constants [G. A. Bain and J. E. Berry *J. Chem. Educ.* 2008, 85, 4, 532]. Magnetic susceptibility data were obtained by measuring the samples' magnetizations between 2 and 300 K in a static applied field of 500 Oe to avoid saturation conditions, and the corresponding susceptibility values were obtained as the ratio of the magnetization with the field. The data around 50 K were removed because they were influenced by the presence of a spurious contribution from oxygen trapped in the SQUID, which generates a peak in that range.

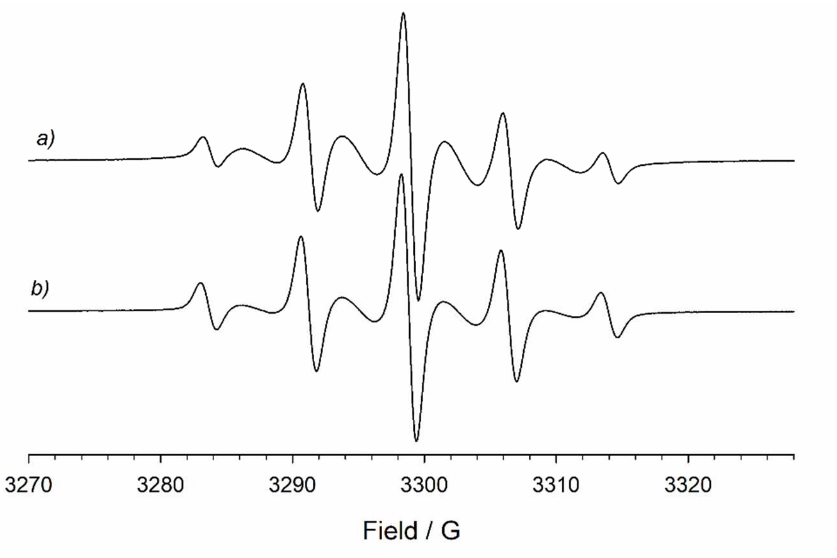

Figure S1. EPR spectra of **Axle-2** (0.3 mM) in MeCN at 323 K in the absence (*a*) and in the presence (*b*) of nine equivalents of TFA.

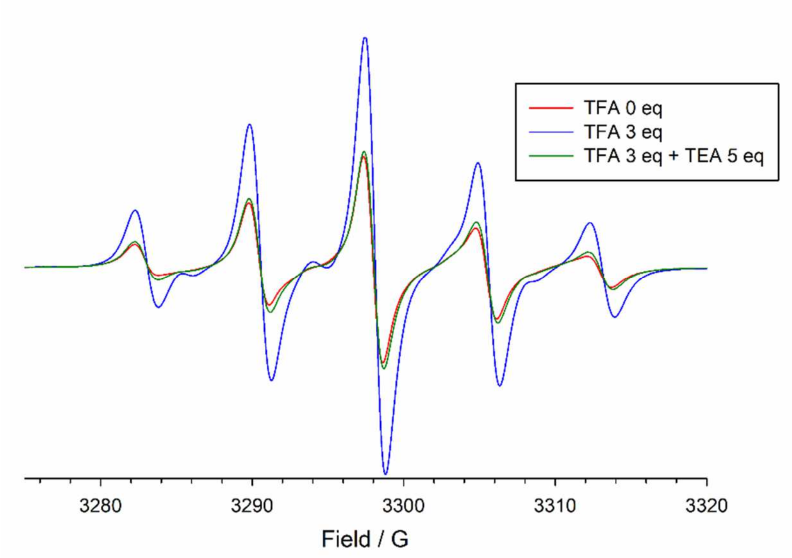

Figure S2. EPR spectra of **Rotax-1** (0.3 mM) in MeCN at 298 K in in the presence of different amounts of trifluoroacetic acid (TFA) and triethylamine (TEA).

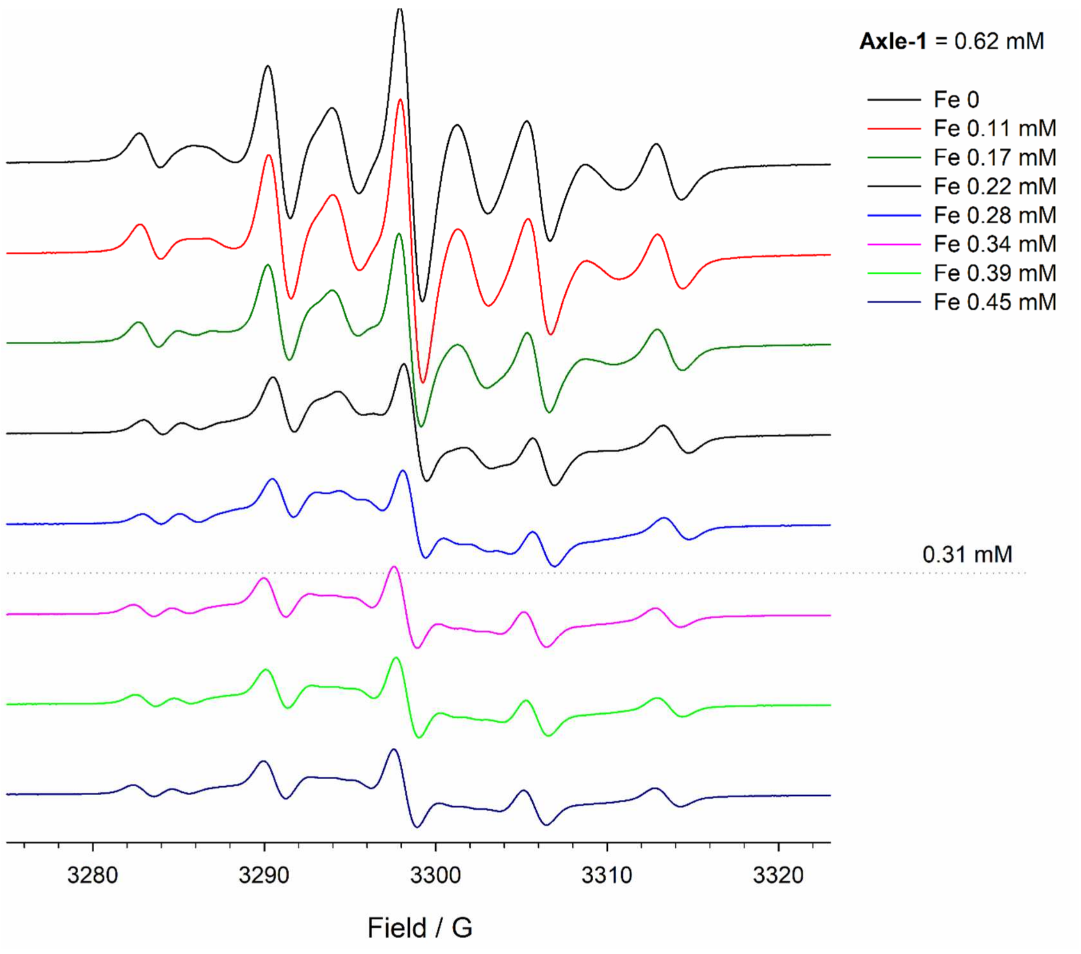

Figure S3. EPR titration of **Axle-1** (0.62 mM) in MeCN at 298 K with $Fe(OTf)_2$.

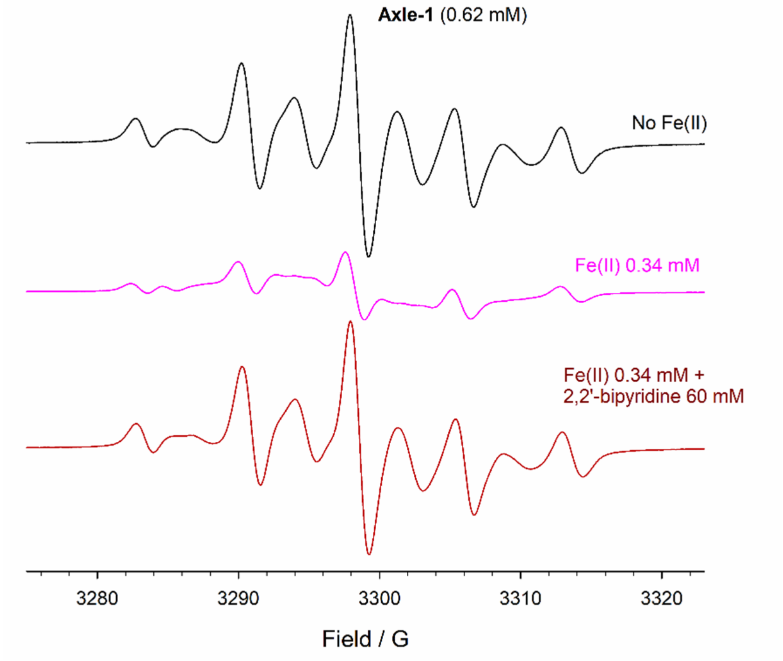

Figure S4. EPR spectra of **Axle-1** (0.62 mM) in MeCN at 298 K in the absence (balck line) and in the presence of 1 eq. of $Fe(OTf)_2$ (purple line) and a large excess of 2,2'-bipyridine (red line).

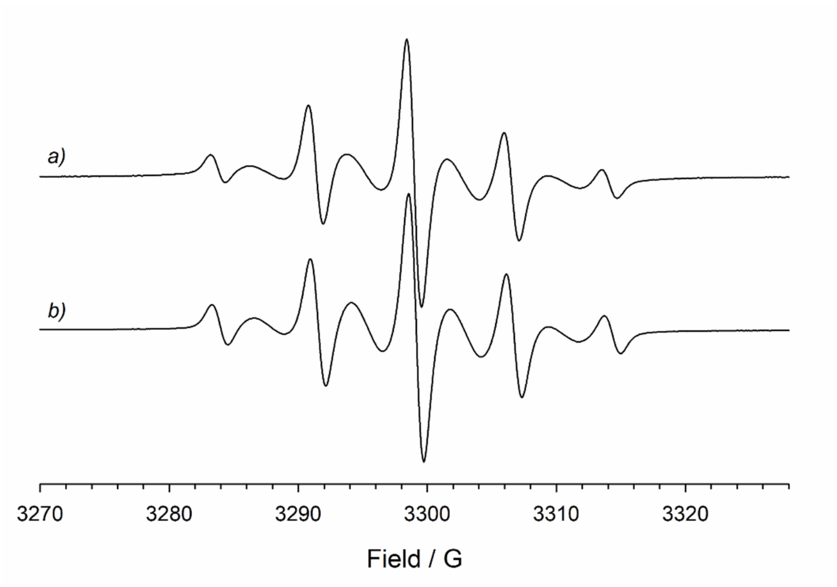

Figure S5. EPR spectra of **Axle-2** (0.3 mM) in MeCN at 323 K in the absence (*a*) and in the presence of half equivalent $Fe(OTf)_2$.

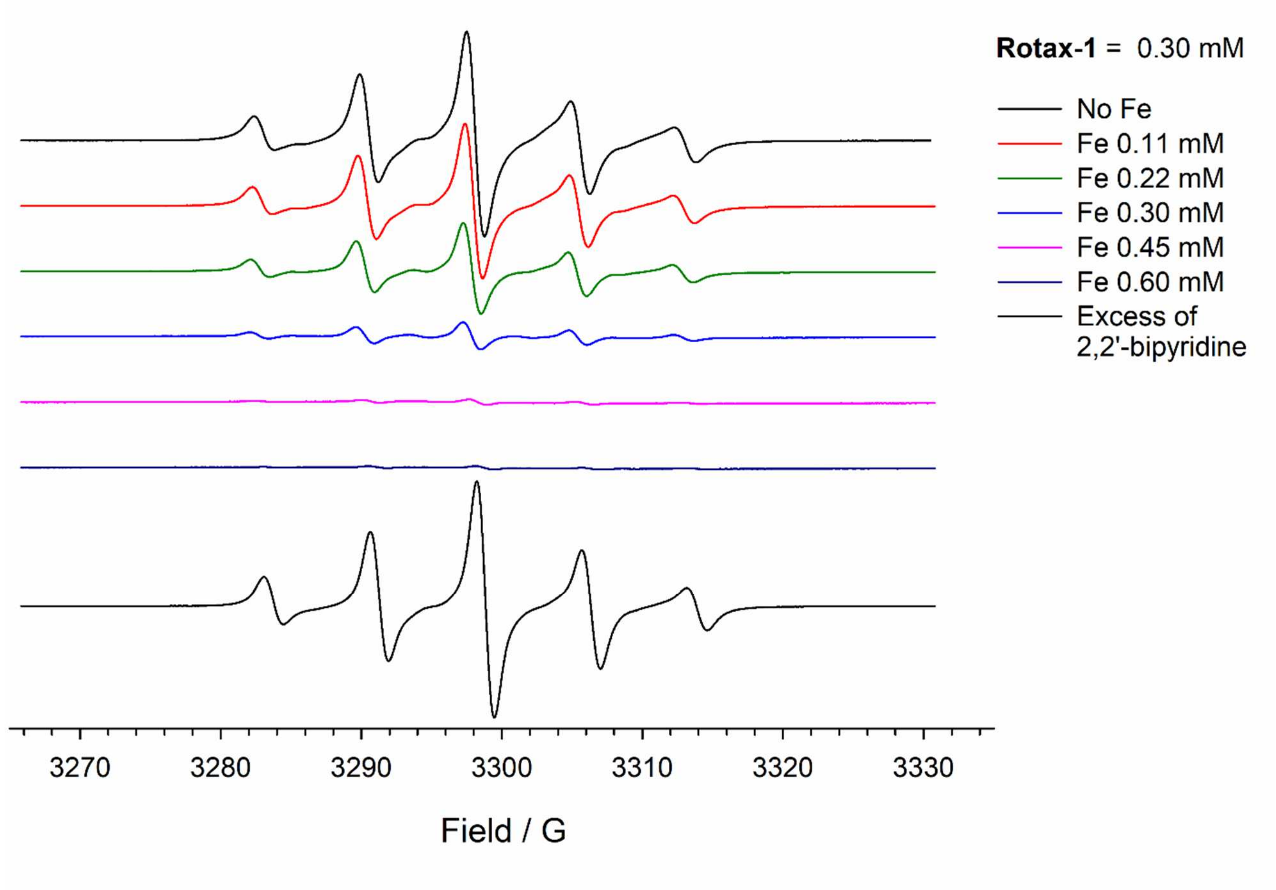

Figure S6. EPR titration of **Rotax-1** (0.3 mM) in MeCN at 298 K with $Fe(OTf)_2$. The spectrum below was recorded after the addition of an excess (100:1) of 2,2'-bipyridine.

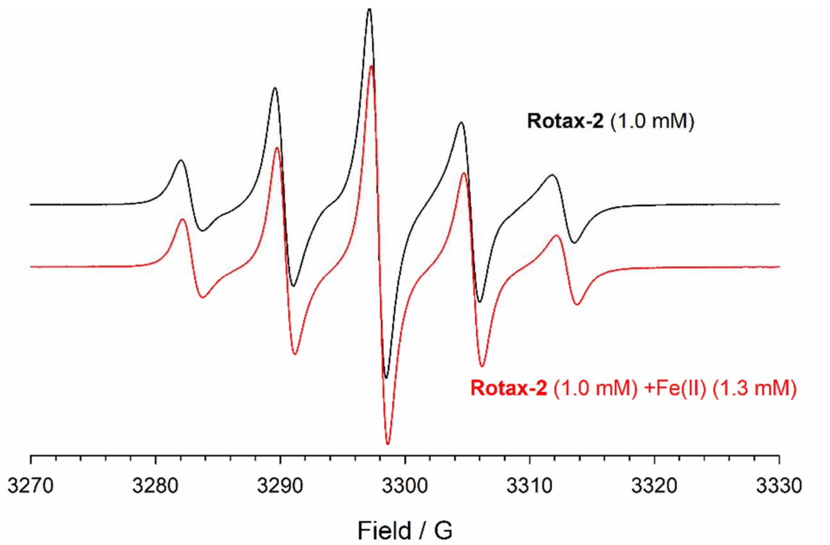

Figure S7. EPR spectra of **Rotax-2** (1.0 mM) in MeCN at 298 K in the absence and in the presence of $Fe(OTf)_2$.

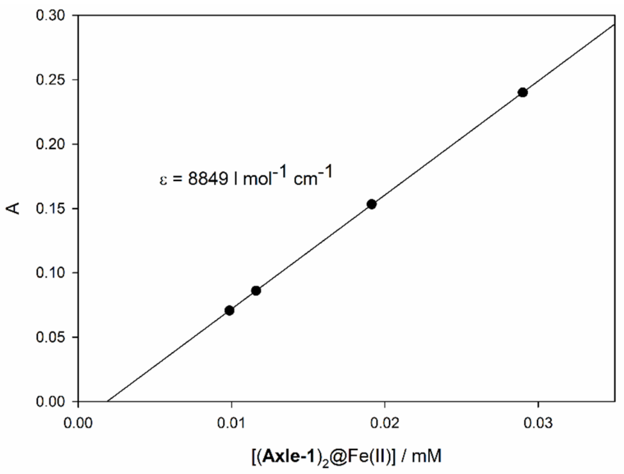

Figure S8. Calibration curve for (**Axle-1**)$_2$@Fe(II) complex concentration vs absorbance at 438 nm in MeCN T= 298 K.

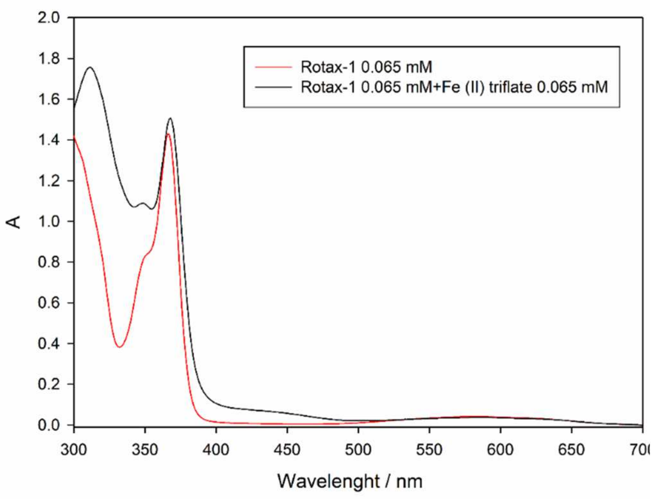

Figure S9. UV-vis absorption spectra of **Rotax-1** in the absence (red line) and in the presence (black line) of 1 eq. of $Fe(OTf)_2$ in MeCN solution at 298 K.

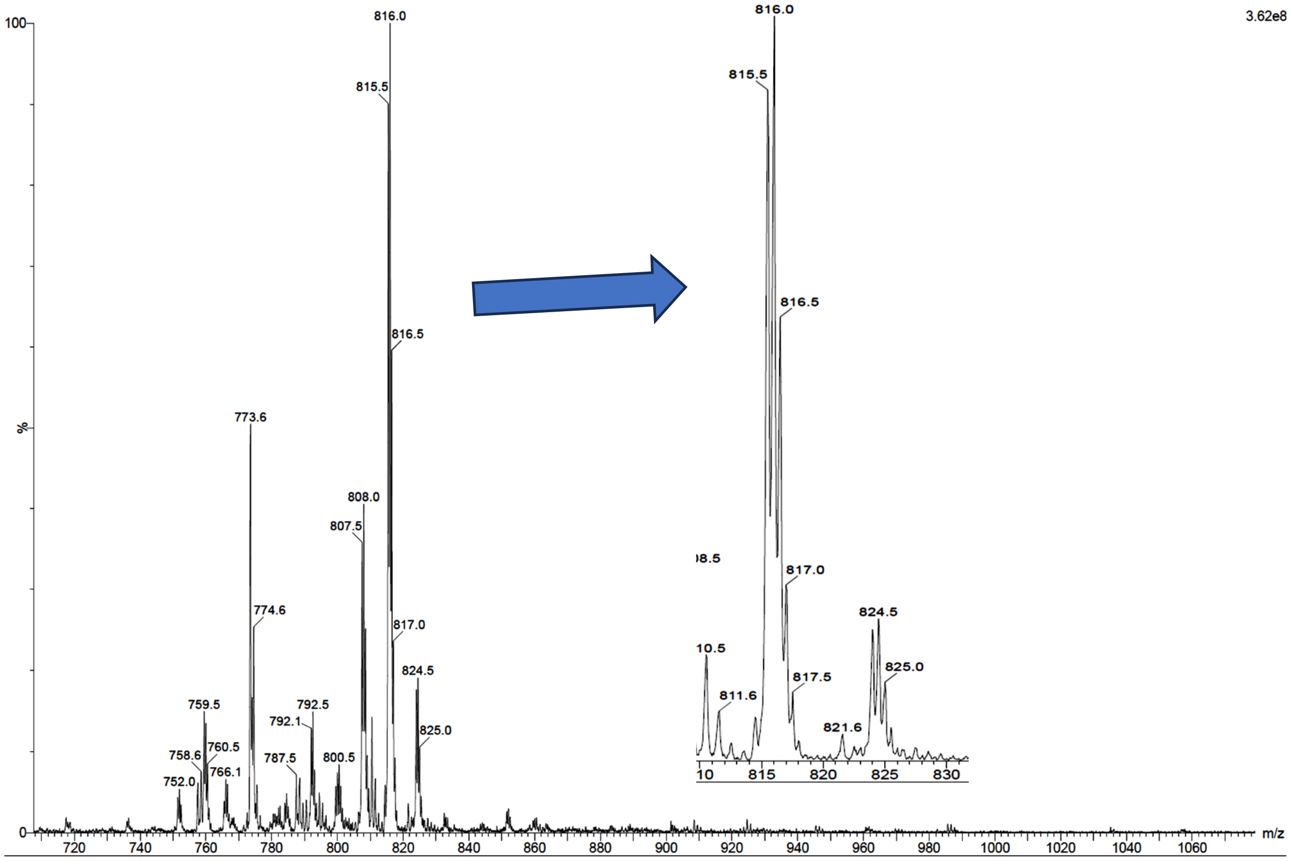

Figure S10. ESI-MS spectrum of **Axle-1** (0.1 mM) in meCN at 298 K in the presence of half equivalent of $Fe(OTf)_2$.

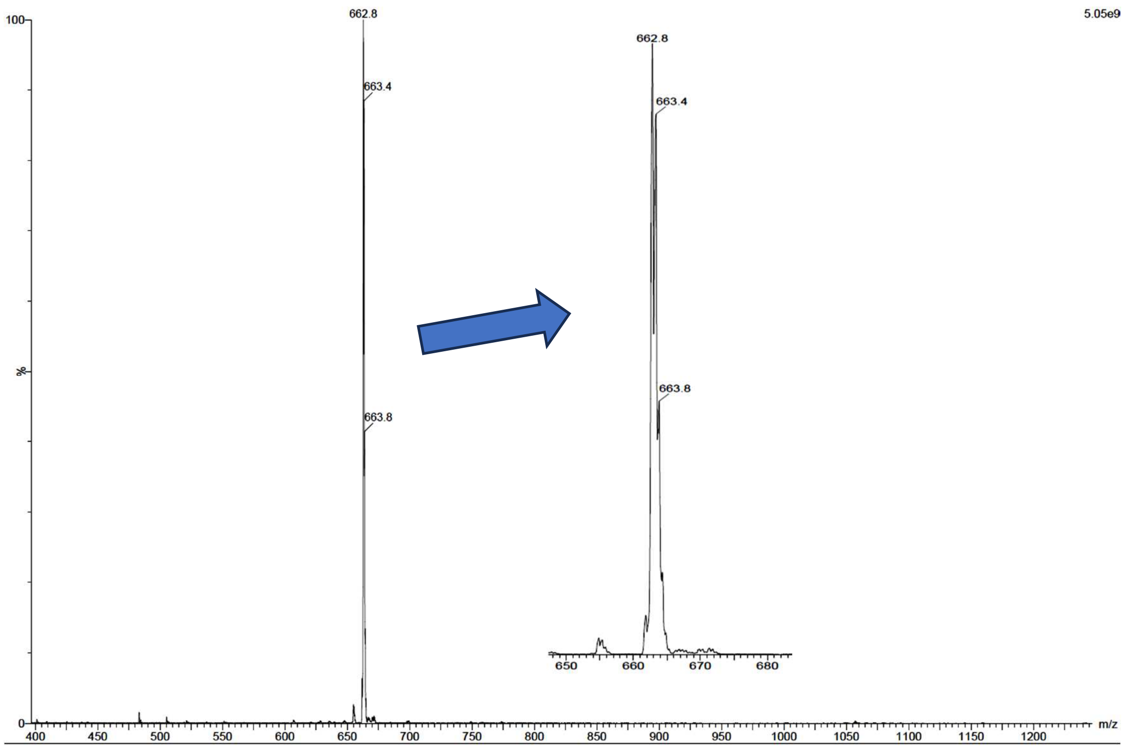

Figure S11. ESI-MS spectrum **Rotax-1** (0.1 mM) in MeCN at 298 K in the presence of one equivalent of $Fe(OTf)_2$

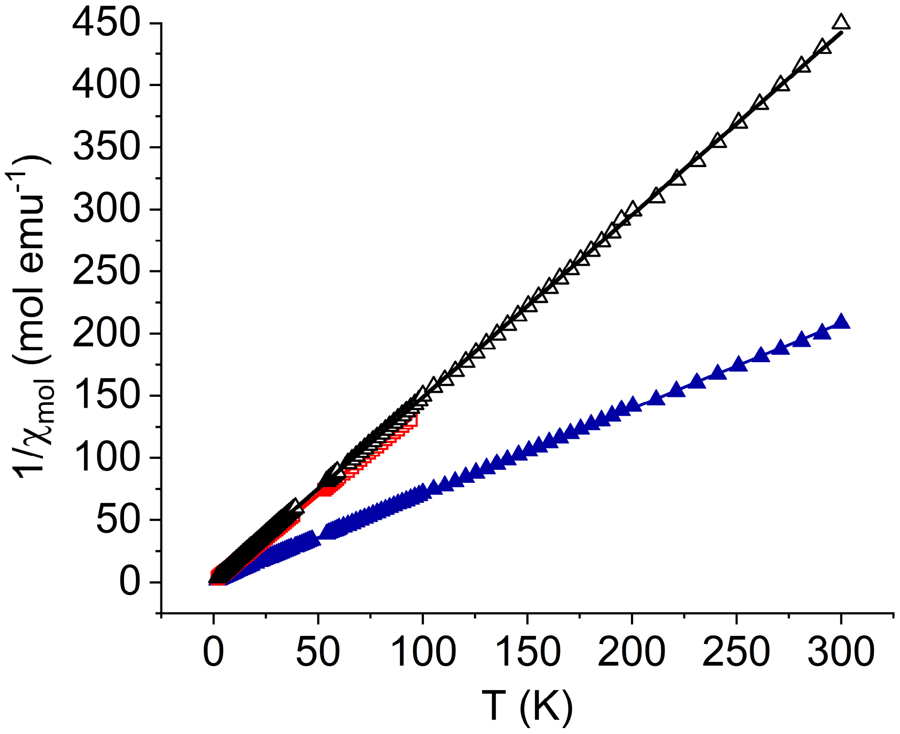

Figure S12. Curie-Weiss plots and corresponding best fit lines obtained using parameters reported in the main text for **Axle-1** (empty triangles), **Rotax-1**(red empty squares) and **Axle-1@Fe**(full blue triangles)

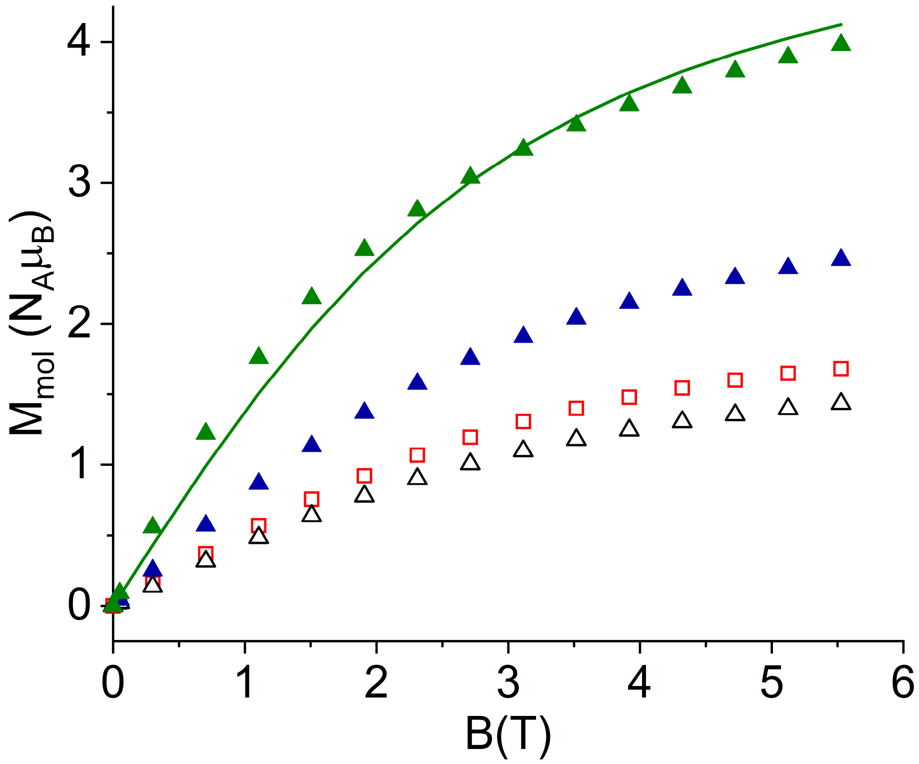

Figure S13. Isothermal magnetization curves, measured at 2 K, for the four investigated derivatives. Green full triangles: **Rotax-1@Fe**; Blue full triangles: **Axle-1@Fe**; Empty red squares: **Rotax-1**; Empti black triangles: **Axle-1.** The continuous line is the best fit to the data for **Rotax-1@Fe** obtained with model and parameters reported in the main text.
